# A non-autonomous optimal control model of renewable energy production under the aspect of fluctuating supply and learning by doing

**DOI:** 10.1007/s00291-016-0444-0

**Published:** 2016-04-27

**Authors:** Elke Moser, Dieter Grass, Gernot Tragler

**Affiliations:** Vienna University of Technology (TU Wien) Institute of Statistics and Mathematical Methods in Economics (SWM), Wiedner Hauptstraße 8/E105-4, 1040 Vienna, Austria

**Keywords:** Optimal control, Nonlinear dynamical systems, Renewable energy, Learning by doing

## Abstract

Given the constantly raising world-wide energy demand and the accompanying increase in greenhouse gas emissions that pushes the progression of climate change, the possibly most important task in future is to find a carbon-low energy supply that finds the right balance between sustainability and energy security. For renewable energy generation, however, especially the second aspect turns out to be difficult as the supply of renewable sources underlies strong volatility. Further on, investment costs for new technologies are so high that competitiveness with conventional energy forms is hard to achieve. To address this issue, we analyze in this paper a non-autonomous optimal control model considering the optimal composition of a portfolio that consists of fossil and renewable energy and which is used to cover the energy demand of a small country. While fossil energy is assumed to be constantly available, the supply of the renewable resource fluctuates seasonally. We further on include learning effects for the renewable energy technology, which will underline the importance of considering the whole life span of such a technology for long-term energy planning decisions.

## Introduction

Facing the impacts of climate change, the rapid economic growth coming along with a higher energy demand as well as the fact that one of the main contributors to the constantly increasing green house gas emissions is given by the energy sector, the possibly biggest problem of this century will be to find a carbon-low, sustainable, and simultaneously secure energy supply. Therefore, the incentives for developing and improving renewable energy technologies have changed during the past decades. Originally, the driving force has been given by the rapidly narrowing horizon of depletion of fossil fuels. However, due to the development of new extraction techniques and the discovery of new sources nowadays, the threats of global warming play a major role. Mitigation policies supporting investments into renewable energy technologies should reduce the emissions and slow down the global warming process. The available alternatives of energy generation in the future, however, strongly depend on structural and technological changes together with the accompanying investment decisions right now, because the development and the diffusion of a new technology is a time-intensive dynamic process (cf. Harmon 2000). This underlines the importance of timely planing for energy technology choices. In contrast to conventional energy generation, renewable energy technologies have high investment costs and, therefore, investment decisions for a new technology are often postponed until they get cheaper. This, however, strongly restricts the scale of alternative energy generation (cf. Berglund and Söderholm 2006; Rong-Gang 2013). Therefore, it is important to consider the whole life span of a new technology for energy planning decisions to include the diffusion process and the cost reduction that come along with implementing the new technology. Another challenge of renewable energy generation is the fact that the supply of renewable sources is not constant at all but fluctuating.

To investigate this issue we consider a small country in which a representative decision maker of the energy sector optimizes a portfolio consisting of fossil and renewable energy. We postulate for simplicity that full information about the energy demand that has to be covered is available and that it is stationary, as done in Coulomb (2011). Instead of assuming that the energy demand depends on the GDP of the country, as done in Chakravorty et al. (2012), or on the electricity price, we follow Messner (1997) and consider the energy demand to be exogenous and, further on, constant. Given this demand and considering the fact that the supply of the used renewable sources is fluctuating seasonally, the representative energy sector decision maker optimizes this portfolio to find the most cost-effective solution. We focus especially on solar energy and follow Chakravorty et al. (2006) in omitting completely the possibility of storage so that the generated energy has to be used immediately or otherwise is lost.

In the literature of recent years, some important developments in macroeconomics and energy economics can be observed, dealing with the issue of technological change. While in some modeling approaches technological change, if considered at all, has been included as an exogenous increase in energy conversion efficiency, more recently the aim has been to model it endogenously, especially in form of learning-by-doing effects which sometimes is also considered as technological learning (see for example Chakravorty et al. 2008, 2011; Köhler et al. 2006; Messner 1997; Reichenbach and Requate 2012). To include the aspects of learning by doing in our model, we use a log-linear learning curve to model decreasing investment costs as a function of accumulated experience.

As we consider in our approach the seasonal fluctuations in the supply of renewable sources, this optimal control problem with one state and two controls exhibits a particular mathematical property by being non-autonomous. Solving this problem by applying Pontryagin’s maximum principle, we are looking for a periodic solution that solves the non-autonomous canonical system, which makes the problem numerically sophisticated and which differs from the usual steady-state analysis of autonomous approaches.

The paper is organized as follows: We briefly present first the concept of learning by doing in energy planing models in Sect. 2. In Sect. 3 we then give a detailed description of the model formulation, while Sect. 4 deals with the solution of the problem. The numerical results are presented and interpreted in Sect. 5. As it turns out that the optimal long-run solution is sensitive with respect to the fossil energy price, the learning coefficient, as well as to geographical site specific parameters, we conduct a sensitivity analysis with respect to these parameters in Sect. 6. Finally, we summarize our findings, give conclusions and a brief outlook on future work in Sect. 7.

## The learning curve concept

The development of the learning curve originates from Wright (1936) who observed that in airplane-manufacturing the number of working-hours spent for the production of an airframe is a decreasing function of the total number of the previously produced airframes of the same type. In other words, this means that the unit costs of labor declined with experience measured in cumulative output. Later, Arrow (1962) used cumulative gross investments in form of cumulative production of capital goods as an index of experience so that each new machine produced and used in the production process changes the production environment and leads to a learning process with continual incentive. There exist some other references in the literature, however, stating that interruptions of the production process could also cause negative learning effects, referred to as forgetting by not doing (e.g. see Argote et al. 1990; Argote and Epple 1990; Benkard 2000; Epple et al. 1991), and, hence, rather net investments are a better index for experience. In all different forms, the learning curve concept has been applied in many fields of research and has become an important tool to measure cost-effectiveness of technologies. Given the goal of achieving adequate technology policies to mitigate climate change, the implementation of endogenous technological change via the learning curve in models of future energy scenarios is essential (e.g. see Gerlagh et al. 2003; Grübler and Messner 1998). The learning curve provides an important tool to measure the cost effectiveness of policy decisions to support new technologies. It connects expected future costs with current investments so that the cost of the new technology depends on earlier developments reflected by the cumulative capacity. This comes along with the path dependence of technological competition.

The learning curve quantifies empirically the impact of learning by doing on the production costs of an industry or a firm by considering the investment costs as a declining function of cumulative capacity or cumulative output. Both of these factors are an approximation of knowledge (cf. Argote et al. 1990). In literature, a variety of different functional forms modeling these interrelationship can be found; the probably most common one, however, is the log-linear function to its simplicity and its observed good fit with data. In this case, the progressive decrease is explained by the so-called progress rate given as

$$\begin{aligned} { PR}= 2^{-\alpha }, \end{aligned}$$

where $\alpha >0$ is the learning coefficient. The progress rate corresponds to the percentage change in costs, when the cumulative capacity is doubled. Therefore, a progress rate of 80$\,\%$ means that the costs are reduced to 80$\,\%$ of its previous value when the cumulative capacity doubles. This reduction of 20$\,\%$ is referred to as the learning-by-doing rate and is given by

$$\begin{aligned} \mathrm{LDR}=1-{ PR}= 1-2^{-\alpha }. \end{aligned}$$

The costs then are calculated as

1 $$\begin{aligned} C_{t}=C_{0}\left( \frac{K_{t}}{K_{0}}\right) ^{-\alpha }, \end{aligned}$$

where $C_{t}$ are the investment costs at time *t*, $K_{t}$ is the cumulative capacity at time *t*, $K_{0}$ is the initial cumulative capacity at time $t=0$, and $C_{0}$ are the initial investment costs. This scaling expresses that for an initially low cumulative capacity, it takes more efforts and investments to produce a given level of energy than for an initially high cumulative capacity (cf. Van der Zwaan et al. 2002). Taking the logarithm of Eq. (1) yields an expression which can be estimated econometrically to get a reasonable value for $\alpha$, and, therefore, for the learning-by-doing rate LDR. This, of course, strongly depends on the type of technology and is crucial for the speed of learning (a survey on estimates of learning rates for a set of energy technologies can be found in McDonald and Schrattenholzer 2001). Equation (1) is also referred to as the single- or one-factor-learning curve. The so-called break-even point is reached, when so much experience is accumulated that the new technology gets competitive with the conventional one.

## The model

To investigate the challenges of including renewable energy into a power system under the aspect of learning by doing, we consider an economy of a small country in which both fossil and renewable energy can be used as perfect substitutes to cover an exogenously given energy demand. Due to the size of the country, we assume that there are no or at least not enough available fossil resources and, therefore, fossil energy has to be imported from other countries for the current market price. As far as renewable energy is concerned, harvesting is for free and the generation is possible within the own country. In contrast to fossil energy, which is assumed to be constantly available, the supply of renewable energy seasonally fluctuates. In order to use this renewable energy resource, capital is necessary for the energy generation for which investments have to be undertaken. We consider for our model a representative energy sector decision maker who chooses the optimal energy portfolio composition for the whole country. It is postulated that this representative energy sector decision maker has full information about the energy demand that has to be covered at each point of time. Therefore, he/she decides on the most cost-effective portfolio consisting of these two energy types, taking into account the seasonal fluctuations of renewable energy supply, the investment costs for renewable energy generation capital which decline with experience, and the import costs of fossil energy. One important implication of the size of the country is that the representative energy sector decision maker is assumed to be a price taker and, therefore, his/her decision has no influence on the market price.

**Fig. 1:**
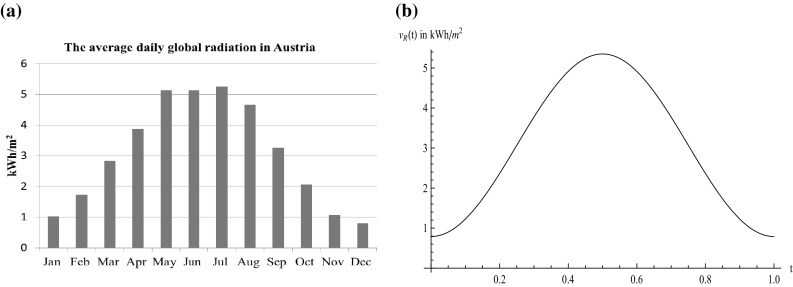
**a** Average global radiation per month in Austria, **b** deterministic function to describe the varying global radiation over 1 year ($t=1$)

We take the considered fossil energy as an aggregate of fossil energy sources (e.g. coal, gas, etc.) and focus on solar energy as renewable resource. To give an example for the seasonal supply of solar energy, Fig. 1a shows the average global radiation per month in Austria. One can clearly observe the seasonal differences that pose a challenge to a constant renewable energy supply over the whole year. Saving, of course, would be supportive in the short-run, but as we rather are interested in long-run solutions and, for this time frame, saving possibilities are limited, we completely omit storage in our model approach and focus only on the change in the portfolio composition. To include these seasonal fluctuations in our model, we use a deterministic time-dependent function

$$\begin{aligned} v_{R}(t)=\nu \sin ^{2}(t\pi )+\tau , \end{aligned}$$

which is plotted in Fig. 1b. The period length of the fluctuation is 1 year, $\tau$ is the minimal supply in winter, and $\nu$ is the maximal increment during summer. To get reasonable parameter values, we have used Austrian data (ZAMG 2012) for estimation. Note that we only consider annual fluctuations and do not include daily fluctuations as well as changes due to weather conditions. To convert solar radiation into energy, specific capital in form of PV cells is necessary. This capital is accumulated by investments $I_{S}(t)$ and depreciates with a factor $\delta _{S}$. The capital accumulation function in our model reads as follows:

$$\begin{aligned} \dot{K_{S}}(t)=I_{S}(t)-\delta _{S} K_{S}(t). \end{aligned}$$

As depreciation is considered in the model, investments $I_{S}(t)$ do not only include acquisition decisions but also maintenance efforts. Later in the paper, this distinction will be important to fully understand the obtained investment decisions. Given the available capital at each point in time and the current supply of global radiation, renewable energy is generated as

$$\begin{aligned} E_{S}\big (t,K_{S}(t)\big )=\big (\nu \sin ^{2}(t\pi )+\tau \big ) K_{S}(t) \eta , \end{aligned}$$

where $\eta$ is the degree of efficiency (cf. Deshmukh and Deshmukh 2008; Nema et al. 2009). For common PV cells that are currently on the market $\eta$ is about 20 %. Note that this function explicitly depends on time *t* which therefore makes the problem non-autonomous.

Since the representative energy sector decision maker is assumed to have exact information about the required energy demand *E*^1^ and no further uncertainties are included, it is postulated that the demand has to be completely satisfied with the portfolio of fossil $E_{F}(t)$ and renewable $E_{S}(t,K_{S}(t))$ energy. Shortfalls are not allowed while surpluses are possible. However, as we do not include the possibility of storage, this implies that surpluses are lost and cannot be further used.^2^ This balance is included in the model by the mixed path constraint

2 $$\begin{aligned} E_{F}(t)+E_{S}\big (t,K_{S}(t)\big )-E\ge 0. \end{aligned}$$

In order to include the aspects of learning by doing, we first make some assumptions about the functional form of the learning curve. While Eq. (1) only is defined for an initial cumulative capital stock of $K_{0}>0$, we enlarge this by allowing also a complete start with renewable energy, meaning $K_{0}=0$. To do so, we follow Berglund and Söderholm (2006), who present a learning curve formula without explicitly modeling the initial cumulative capital. Further on, we add an additional term $\epsilon$ defining the initial investment costs when the cumulative capital stock is zero, as done in Hartley et al. (2010). The new learning curve then reads as

$$\begin{aligned} C_{t}=C_{0}(K_{S}(t)+\epsilon )^{-\alpha }, \end{aligned}$$

where the initial investment costs are given as

$$\begin{aligned} C_{0}=I_{S}(t)\Big (b+cI_{S}(t)\Big ). \end{aligned}$$

Note that we distinguish between linear investment and quadratic adjustment costs, where the latter ones arise from installation efforts (cf. Feichtinger et al. 2006; Rasmussen 2001). The specification of the learning curve implies that a rapid increase in the renewable energy capital stock is costly, which is relevant for the speed of the economy’s switch to renewable energy generation (cf. Rasmussen 2001). Given the current market price for fossil energy ${{ p}_{ F}}$, the representative energy sector decision maker determines the most cost-effective solution by minimizing total expenditures given by investment costs in renewable energy capital and import costs for fossil energy. Hence, the total costs read as

$$\begin{aligned} C_{t}=I_{S}(t)\left( b+cI_{S}(t)\right) \left( K_{S}(t)+\epsilon \right) ^{-\alpha }+{{ p}_{ F}}E_{F}(t). \end{aligned}$$

Summing up, we consider a non-autonomous optimal control model with infinite horizon, two controls representing the capital investments and the imported fossil energy, and one state describing the capital stock. This cost minimization problem is transformed to the equivalent maximization problem

3 $$\begin{aligned} \max _{E_{F}(t), I_{S}(t)} \int _{0}^{\infty }e^{-rt}\bigg (-I_{S}(t)\left( b+cI_{S}(t)\right) \left( K_{S}(t)+\epsilon \right) ^{-\alpha }-{{ p}_{ F}}E_{F}(t)\bigg )\mathrm{d}t \end{aligned}$$

3a $$\begin{aligned} \text {s.t.:}\quad&\dot{K_{S}}(t)=I_{S}(t)-\delta _{S} K_{S}(t),\end{aligned}$$

3b $$\begin{aligned}&E_{F}(t)+E_{S}\big (t,K_{S}(t)\big )-E\ge 0,\end{aligned}$$

3c $$\begin{aligned}&E_{S}\big (t,K_{S}(t)\big )=\big (\nu \sin ^{2}(t\pi )+\tau \big ) K_{S}(t) \eta ,\end{aligned}$$

3d $$\begin{aligned}&E_{F}(t), I_{S}(t)\ge 0. \end{aligned}$$

## Solution

### Canonical system and necessary first order conditions

Let $(K_{S}^{*}(t),E_{F}^{*}(t)),I_{S}^{*}(t),$ be an optimal solution of the control problem in (3); then, according to Pontryagin’s maximum principle for infinite time horizon problems (cf. Grass et al. 2008), there exists a continuous and piecewise continuous differentiable function $\lambda (t)\in \mathbb {R}$ and a constant $\lambda _{0}\ge 0$ satisfying for all $t\ge 0$ that

$$\begin{aligned} (\lambda _{0},\lambda (t))\ne & {} 0,\\ \mathscr {H}(K_{S}^{*}(t),E_{F}^{*}(t),I_{S}^{*}(t),\lambda (t),\lambda _{0},t)= & {} \max _{E_{F}(t), I_{S}(t) \in \Omega }\mathscr {H}(K_{S}^{*}(t),E_{F}(t),I_{S}(t),\lambda (t),\lambda _{0},t), \end{aligned}$$

where $\mathscr {H}$ defines the *current-value Hamiltonian*^3^ which reads as

$$\begin{aligned} \mathscr {H}(K_{S},E_{F},I_{S},\lambda ,\lambda _{0},t)= & {} \lambda _{0}\left( -(bI_{S}(t)+cI_{S}(t)^{2})(K_{S}(t)+\epsilon )^{-\alpha }-{{ p}_{ F}}E_{F}(t)\right) \\&\quad +\lambda (t)(I_{S}(t)-\delta _{S}K_{S}(t)), \end{aligned}$$

and $\Omega$ is the feasible region determined by the inequality constraints (3b) and (3d). To analyze this model, we, therefore, consider the *Lagrangian* (augmented current-value Hamiltonian) which reads as

$$\begin{aligned}&\mathscr {L}(K_{S},E_{F},I_{S},\lambda ,\lambda _{0},\mu _{1},\mu _{2},\mu _{3},t)\\&\quad =\lambda _{0}\left( -\left( bI_{S}(t)+cI_{S}(t)^{2}\right) (K_{S}(t)+\epsilon )^{-\alpha }-{{ p}_{ F}}E_{F}(t)\right) +\lambda (t)(I_{S}(t)-\delta _{S}K_{S}(t))\\&\qquad +\mu _{1}(t)\left( E_{F}(t)+\left( \nu \sin ^{2}(t\pi )+\tau \right) K_{S}(t)\eta -E\right) +\mu _{2}(t)E_{F}(t)+\mu _{3}(t)I_{S}(t), \end{aligned}$$

where $\mu _{1}(t),\mu _{2}(t)$ and $\mu _{3}(t)$ are the Lagrange multipliers for the mixed path constraint and the non-negativity conditions, respectively. Further on, at each point where the controls are continuous,

$$\begin{aligned} \dot{\lambda }(t)=r\lambda (t)-\frac{\partial \mathscr {L}(K_{S},E_{F},I_{S},\lambda ,\lambda _{0},\mu _{1},\mu _{2},\mu _{3},t)}{\partial K_{S}} \end{aligned}$$

is given and the complementary slackness conditions,

$$\begin{aligned}&\displaystyle \mu _{1}(t)\left( E_{F}^{*}(t)+E_{S}^{*}\big (t,K_{S}^{*}(t)\big )-E\right) =0,\quad \mu _{1}(t)\ge 0,\\&\displaystyle \mu _{2}(t)E_{F}^{*}(t)=0,\quad \mu _{2}(t)\ge 0,\\&\displaystyle \mu _{3}(t)I_{S}^{*}(t)=0,\quad \mu _{3}(t)\ge 0, \end{aligned}$$

have to hold. Further on, we require the limiting transversality condition

$$\begin{aligned} \lim _{t\rightarrow \infty }\lambda (t) e^{-rt}=0, \end{aligned}$$

to be satisfied. It can be proven that without loss of generality we can set for the subsequent analysis $\lambda _{0}=1$. The necessary first-order conditions and the adjoint equation then are given as follows:

$$\begin{aligned}&\displaystyle \frac{\partial \mathscr {L}}{\partial E_{F}(t)}=-{{ p}_{ F}}+\mu _{1}(t)+\mu _{2}(t)=0,\\&\displaystyle \frac{\partial \mathscr {L}}{\partial I_{S}(t)}=-b(K_{S}(t)+\epsilon )^{-\alpha }-2cI_{S}(t)(K_{S}(t)+\epsilon )^{-\alpha }+\lambda (t)+\mu _{3}(t)=0, \\&\displaystyle \Leftrightarrow I_{S}(t)=\frac{(K_{S}(t)+\epsilon )^{\alpha }(\lambda (t)+\mu _{3}(t))-b}{2c},\nonumber \\&\displaystyle \dot{\lambda }(t)=\lambda (t) r-\frac{\partial \mathcal {L}}{\partial K_{S}(t)}=(r+\delta _{S})\lambda (t)-\alpha (b+cI_{S}(t))I_{S}(t)(K_{S}(t)+\epsilon )^{-\alpha -1}\nonumber \\&\displaystyle -\mu _{1}(t)\eta (\nu \sin ^{2}(t\pi )+\tau ), \end{aligned}$$

which yields the canonical system as

4 $$\begin{aligned} \dot{K}_{S}(t)= & {} \frac{(K_{S}(t)+\epsilon )^{\alpha }(\lambda (t)+\mu _{3}(t))-b}{2c}-\delta _{S}K_{S}(t)=:f^{K_{S}}(t, K_{S}(t),\lambda (t)),\nonumber \\ \end{aligned}$$

5 $$\begin{aligned} \dot{\lambda }(t)= & {} \alpha (K_{S}(t)+\epsilon )^{-\alpha -1}\left( \frac{b^{2}-(K_{S}(t)+\epsilon )^{2\alpha }(\lambda (t)+\mu _{3}(t))^{2}}{4c}\right) \nonumber \\&-{{ p}_{ F}}\eta (\nu \sin ^{2}(t\pi )+\tau )+\lambda (t)(r+\delta _{S})=:f^{\lambda }(t, K_{S}(t),\lambda (t)). \end{aligned}$$

One can easily show that a solution path within the boundaries of the model, meaning that both controls are positive and the mixed-path constraint of (2) is satisfied with inequality, never can be optimal. The reason lies within the linearity of the Lagrangian in $E_{F}(t)$ and that the partial derivative of the Lagrangian with respect to $E_{F}(t)$ is negative, which yields a bang–bang solution where the maximum is reached at the lowest feasible control $E_{F}(t)$. Hence, the cost of inefficient surpluses could immediately be reduced by decreasing the amount of fossil energy until either, the mixed path constraint is satisfied with equality, or the fossil energy amount gets zero, which both correspond to boundary cases. Therefore, we can completely omit the inner solution and focus for the following analysis on the feasible boundaries. In total, we can distinguish between three of them, the fossil case with no investments in renewable energy capital, $E_{F}(t)>0$, $I_{S}(t)=0$ and $E_{F}(t)+E_{S}(t,K_{S}(t))-E=0$,^4^ the mixed case where both types of energy are used for the coverage with $E_{F}(t)$, $I_{S}(t)>0$ and $E_{F}(t)+E_{S}(t,K_{S}(t))-E=0$, and the renewable case, where no more fossil energy is used in addition to renewable energy to cover the demand, meaning that $E_{F}(t)=0$, $I_{S}(t)>0$, and $E_{S}(t,K_{S}(t))-E\ge 0$ holds. Inserting the corresponding values for the Lagrange multipliers yields the three different canonical systems, with the fossil case as

6 $$\begin{aligned}&\displaystyle \dot{K_{S}}(t)=-\delta _{S}K_{S}(t),\end{aligned}$$

7 $$\begin{aligned}&\displaystyle \dot{\lambda }(t)=\lambda (t)(r+\delta _{S})-{{ p}_{ F}}\eta (\nu \sin ^{2}(t\pi )+\tau ), \end{aligned}$$

the mixed case as

8 $$\begin{aligned}&\displaystyle \dot{K_{S}}(t)=\frac{\lambda (t)(K_{S}(t)+\epsilon )^{\alpha }-b}{2c}-\delta _{S}K_{S}(t),\end{aligned}$$

9 $$\begin{aligned}&\displaystyle \dot{\lambda }(t)=\alpha (K_{S}(t)+\epsilon )^{-\alpha -1}\left( \frac{b^{2}-(K_{S}(t)+\epsilon )^{2\alpha }\lambda (t)^{2}}{4c}\right) \nonumber \\&\displaystyle -{{ p}_{ F}}\eta (\nu \sin ^{2}(t\pi )+\tau )+\lambda (t)(r+\delta _{S}), \end{aligned}$$

and the renewable case as

10 $$\begin{aligned}&\displaystyle \dot{K_{S}}(t)=\frac{\lambda (t)(K_{S}(t)+\epsilon )^{\alpha }-b}{2c}-\delta _{S}K_{S}(t),\end{aligned}$$

11 $$\begin{aligned}&\displaystyle \dot{\lambda }(t)=\alpha (K_{S}(t)+\epsilon )^{-\alpha -1}\left( \frac{b^{2}-(K_{S}(t)+\epsilon )^{2\alpha }\lambda (t)^{2}}{4c}\right) +\lambda (t)(r+\delta _{S}). \end{aligned}$$

### Periodic solution

As the canonical system in (4) and (5) is not only non-autonomous, but in addition also periodic with period length 1, it, therefore, belongs to a special class of non-autonomous differential equation systems, also called one-periodic differential equations. Consequently, if *x*(*t*) is a solution of the canonical system, also $x(t+k)$ for every integer *k* is a solution. Due to this periodicity in the dynamics, a candidate for the optimal long-run solution of the problem in (3), which is the solution to which each optimal solution is converging to over time, is given by a periodic solution with the period length of 1 year. In order to find such candidates, we first determine the instantaneous equilibrium points (cf. Ju et al. 2003), which are calculated for the general canonical system in (4) and (5) as the solution of the differential equation system

$$\begin{aligned} \dot{K}_{S}(t)= & {} f^{K_{S}}(t, K_{S}^{IEP}(t),\lambda ^{IEP}(t))=0, \\ \dot{\lambda }(t)= & {} f^{\lambda }(t, K_{S}^{IEP}(t),\lambda ^{IEP}(t))=0. \end{aligned}$$

To find the periodic solutions of this model, we then use these instantaneous equilibrium points as starting solution for the boundary value problem

$$\begin{aligned} \dot{K_{S}}(t)= & {} f^{K_{S}}(t, K_{S}(t),\lambda (t)), \quad \text { with } K_{S}(1)=K_{S}(0),\\ \dot{\lambda }(t)= & {} f^{\lambda }(t, K_{S}(t),\lambda (t)),\quad \text { with } \lambda (1)=\lambda (0), \end{aligned}$$

which yields the periodic solution $\left( K_{S}^{*}(t),\lambda ^{*}(t)\right)$ that lies completely within one of the three boundary cases. However, it can happen that the solution at some point leaves the current feasible boundary before the course of the period of 1 year is run through. In this case one cannot find a closed periodic solution within this feasible area and one has to switch to the corresponding canonical system to get a periodic solution existing of several arcs. Therefore, a multi-point boundary value problem has to be solved. At each point of time where the constraints of the current region are violated, a switch to the proper region happens, meaning that the according canonical system is used to continue the solution. For *n* switching times $\tau _{1},\dots \tau _{n}$, which satisfy

$$\begin{aligned} \tau _{0}:=0<\tau _{1}<\tau _{2}< \dots <\tau _{n-1}<\tau _{n}<1=:\tau _{n+1}, \end{aligned}$$

$n+1$ arcs have to be calculated for which the continuity at each switching time has to be guaranteed. We introduce an index

$$\begin{aligned} a_{i}={\left\{ \begin{array}{ll} 1,&{}\text { for the fossil region},\\ 2,&{}\text { for the mixed region},\\ 3,&{}\text { for the renewable region}, \end{array}\right. } \end{aligned}$$

that distinguishes the canonical systems for the three boundary cases described in (6)–(11) for each arc *i* with $i=1,\dots ,n+1$. For the numerical solution of the system, for each arc $i+1$ we use the time transformation

$$\begin{aligned} T(s)=(\tau _{i}-\tau _{i-1})(s-i)+\tau _{i} \end{aligned}$$

so that it can be solved with fixed time intervals $[i-1,i]$. We then have to solve for $i=1,\dots ,n+1$, $j=1,\dots ,n$, $s \in [i-1,i]$, and the switching times $\tau _{i}$ with $\tau _{0}=0$, $\tau _{n+1}=1$ the multi-point boundary problem

12 $$\begin{aligned}&\displaystyle \dot{K}_{S_{i}}(s)=(\tau _{i}-\tau _{i-1})f^{K_{S}}_{a_{i}}(T(s), K_{S_{i}}(s),\lambda _{i}(s)),\end{aligned}$$

13 $$\begin{aligned}&\displaystyle \dot{\lambda }_{i}(s)=(\tau _{i}-\tau _{i-1})f^{\lambda }_{a_{i}}(T(s), K_{S_{i}}(s),\lambda _{i}(s)), \end{aligned}$$

14 $$\begin{aligned}&\displaystyle \left( K_{S_{j}}(\tau _{j}),\lambda _{j}(\tau _{j})\right) =\left( K_{S_{j+1}}(\tau _{j}),\lambda _{j+1}(\tau _{j})\right) ,\end{aligned}$$

15 $$\begin{aligned}&\displaystyle \left( K_{S_{n}}(1),\lambda _{n}(1)\right) =\left( K_{S_{1}}(0),\lambda _{1}(0)\right) ,\end{aligned}$$

16 $$\begin{aligned}&\displaystyle c(a_{j},a_{j+1})=0. \end{aligned}$$

Equations (14) and (15) ensure that the continuity in state and controls at each switch is given and, as a periodic solution is calculated, the beginning and the endpoint coincide. Equation (16) finally guarantees the necessary condition that the Lagrangian is continuous as well. This condition is dependent on the involved regions as well as on the direction of the switch and is given for $j=1,\dots ,n$ as

$$\begin{aligned} c(a_{j},a_{j+1})={\left\{ \begin{array}{ll} (K_{S_{j}}(\tau _{j})+\epsilon )^{\alpha }\lambda _{j}(\tau _{j})-b=0,&{}\text { if }\{a_{j},a_{j+1}\}\in \{\{1,2\},\{2,1\}\},\\ E_{S}(\tau _{j},K_{S_{j}}(\tau _{j}))-E=0,&{}\text { if } \{a_{j},a_{j+1}\}\in \{\{2,3\},\{3,2\}\}. \end{array}\right. } \end{aligned}$$

### Stability

In order to analyze the dynamic behavior of an obtained periodic solution $\Gamma (t)$ of the canonical system (4) and (5) with period length 1, we calculate the monodromy matrix as the principal matrix solution of the variational equation

$$\begin{aligned} \dot{y}= & {} J(t) y,\\ y(0)= & {} \begin{pmatrix}1&{}\quad 0\\ 0&{}\quad 1\end{pmatrix}\nonumber , \end{aligned}$$

where *J*(*t*) is the Jacobian matrix evaluated at the periodic solution $\Gamma (t)$,

$$\begin{aligned} J(t)=\begin{pmatrix}\frac{\partial f^{K_{S}}}{\partial K_{S}}&{}\quad \frac{\partial f^{K_{S}}}{\partial \lambda }\\ \frac{\partial f^{\lambda }}{\partial K_{S}}&{}\quad \frac{\partial f^{\lambda }}{\partial \lambda }\end{pmatrix}\left( \Gamma (t)\right) . \end{aligned}$$

Determining the Jacobian matrix for the fossil case yields

$$\begin{aligned} J(t) =\begin{pmatrix}-\delta _{S}&{}\quad 0\\ 0&{}\quad r+\delta _{S}\end{pmatrix}, \end{aligned}$$

and hence the monodromy matrix

17 $$\begin{aligned} M=e^{J(1)}=\begin{pmatrix}e^{-\delta _{S}}&{}\quad 0\\ 0&{}\quad e^{r+\delta _{S}}\end{pmatrix}, \end{aligned}$$

with the eigenvalues

18 $$\begin{aligned} e_{1}=e^{-\delta _{S}},\quad e_{2}=e^{r+\delta _{S}}. \end{aligned}$$

The eigenvalues of the monodromy matrix reflect the stability of the periodic solution. Let $e_{i}$, $i=1,\dots ,n$ be the eigenvalues of the monodromy matrix and let

19 $$\begin{aligned} n^{+}:=\{i:|e_{i}|<1\},\quad n^{-}:=\{i:|e_{i}|>1\} \end{aligned}$$

be the sets of the stable ($n^{+}$) and unstable ($n^{-}$) eigenvalues; a periodic solution $\Gamma (t)$ is called of saddle-type if

20 $$\begin{aligned} |n^{+}| |n^{-}|>0 \end{aligned}$$

holds, which means that at least one of each type has to exist. If $|n^{-}|=0$, the periodic solution is unstable (see Grass et al. 2008). Further on, if no eigenvalue $e_{i}=1, i \in \{1,\dots ,n\}$ occurs, it even is a hyperbolic cycle which guarantees that the behavior of the system near this periodic solution can be fully described by its linearisation (see Hale and Koçak 1991). As $0<\delta _{S}<1$ and $r+\delta _{S}>0$ always holds, this implies that every fossil solution that can be found is of saddle-type.^5^ Calculating the Jacobian matrix for the mixed and the renewable case yields

$$\begin{aligned} J(t) =\begin{pmatrix}-\delta _{S}+\frac{\alpha (K_{S}(t)+\epsilon )^{\alpha -1}\lambda (t)}{2c}&{} \frac{(K_{S}(t)+\epsilon )^{\alpha }}{2c}\\ \\ -\frac{\alpha (K_{S}(t)+\epsilon )^{-\alpha -2}\left( b^2(1+\alpha )+(\alpha -1)(K_{S}(t)+\epsilon )^{2\alpha }\lambda ^{2}\right) }{4c} &{} \qquad r+\delta _{S}-\frac{\alpha (K_{S}(t)+\epsilon )^{\alpha -1}\lambda (t)}{2c}\end{pmatrix}. \end{aligned}$$

Note that here the Jacobian matrix and, therefore, also the monodromy matrix depend on the periodic solution $\Gamma (t)$. Consequently, a general statement on the stability of the mixed and renewable periodic solutions is not possible.

### Numerical continuation of optimal paths

In order to calculate a trajectory starting at an initial capital stock $K_{0}$ and leading into the optimal long-run periodic solution that completely lies within one of the feasible boundary regions, one has to solve for $t\in [0,T]$ the boundary value problem

21 $$\begin{aligned}&\displaystyle \dot{K_{S}}(t)=f^{K_{S}}(t, K_{S}(t),\lambda (t)),\end{aligned}$$

22 $$\begin{aligned}&\displaystyle \dot{\lambda }(t)=f^{\lambda }(t,\lambda (t)),\end{aligned}$$

23 $$\begin{aligned}&\displaystyle K_{S}(0)=K_{0},\end{aligned}$$

24 $$\begin{aligned}&\displaystyle 0=F'\left( \begin{pmatrix} K_{S}(T)\\ \lambda (T)\end{pmatrix}-\begin{pmatrix} K_{S}^{*}(0)\\ \lambda ^{*}(0)\end{pmatrix} \right) , \end{aligned}$$

where the matrix *F* is spanning the orthogonal complement to the stable eigenspace (see Grass 2012) and *T* is the truncation time of the path. The condition in (24) guarantees that the solution ends on the linearized stable manifold to which the vector *F* is orthogonal (for a more detailed analysis of the so-called asymptotic boundary condition see Lentini and Keller 1980).

**Table 1:** Parameter values used for the numerical analysis

Interpretation	Parameter	Value
Investment costs	*b*	0.6
Adjustment costs	*c*	0.3
Energy demand	*E*	2000
Fossil energy price	${{ p}_{ F}}$	0.051
Discount rate	*r*	0.04
Learning coefficient	$\alpha$	0.25
Depreciation rate	$\delta _{S}$	0.03
Initial investment costs	$\epsilon$	1
Degree of efficiency	$\eta$	0.2
Maximal radiation increment	$\nu$	4.56
Minimal radiation in winter	$\tau$	0.79

## Results

**Fig. 2:**
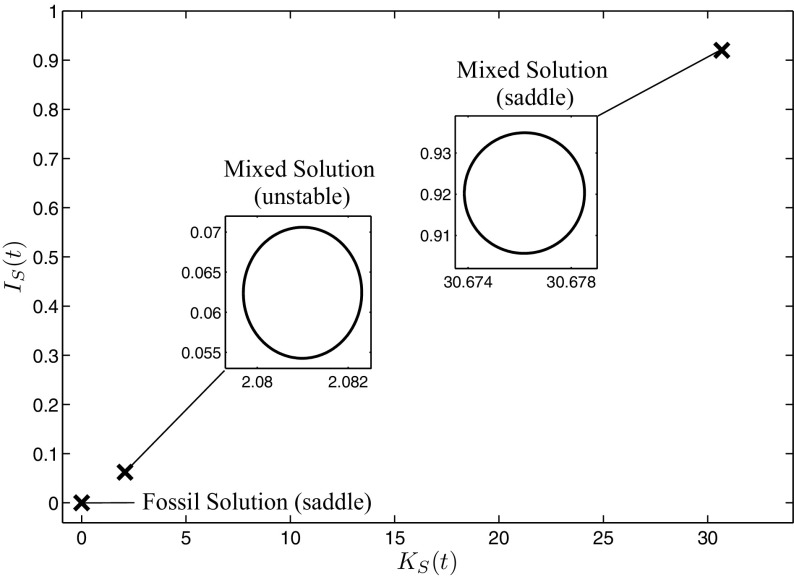
The three detected periodic solutions in the state-control space

We set for the following analysis the parameters as summarized in Table 1: Solving the canonical system for these parameters yields three periodic solutions, where one belongs to the fossil case with zero investments $I_{S}(t)=0$ and a fossil energy amount $E_{F}(t)=E$, and the two other ones correspond to the mixed case with both controls greater than zero. As we have shown analytically in Eqs. (17) and (18), the fossil solution always is of saddle-type. To investigate the stability of the other two mixed solutions, we calculate the eigenvalues of the monodromy matrix, which shows that the lower mixed solution is an unstable focus, while the higher one is also of saddle-type. The three solutions are shown in Fig. 2 and, together with their eigenvalues, are summarized in Table 2.

**Table 2:** Multiple periodic solutions for ${{ p}_{ F}} = 0.051$

Solution	$K_{S}^{*}(0)$	$I_{S}^{*}(0)$	$E_{F}^{*}(0)$	Eigenvalues	Objective function (in $10^3$)
Fossil	0.0000	0.0000	2000.00	{0.9704, 1.0725}	$-$2.4500
Mixed low	2.0797	0.0623	1999.67	{1.0182+0.0645i,	$-$2.4491
				1.0182-0.0645i}	
Mixed high	30.6739	0.9201	1995.15	{0.9827, 1.0591}	$-$2.4351

**Fig. 3:**
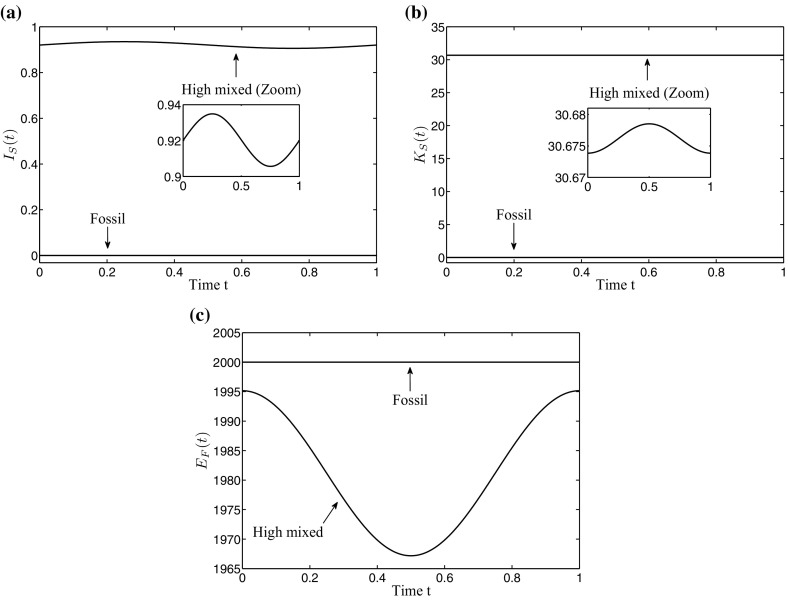
Time-control paths for the three detected periodic solutions: **a** renewable energy investments $I_{S}(t)$, **b** renewable energy capital $K_{S}(t)$, and **c** fossil energy $E_{F}(t)$

The time-control paths and the time-state paths of the two saddle-type solutions are shown in more detail in Fig. 3. To correctly understand the fluctuating investment path shown in Fig. 3a, it is important to distinguish between acquisition investments and maintenance investments. Remember that we have included depreciation in the state equation in (3a) and, consequently, maintenance investments are necessary to keep the capital in a good condition. Therefore, along a path leading into a long-run periodic solution, both, acquisition investments for new capital as well as maintenance investments for the already accumulated capital, are necessary to increase the capital stock. In the periodic solution itself, however, the desired capital stock level is already reached and only maintenance investments are required. While in the fossil solution no maintenance investments $I_{S}(t)$ are made, one can see their seasonality for the high mixed solution. Their fluctuations result from the time lag between the depreciation process and the optimal timing of maintenance investments, as the incentive for maintaining the capital is higher shortly before the global radiation peak in summer. Therefore, the investment decision of the high mixed solution in Fig. 3a can be interpreted as follows: In winter, the global radiation is weak. Consequently, the benefit of the capital stock with respect to renewable energy generation and, hence, the incentive for high maintenance investments is low. For this reason, investments are kept on a low level. However, as soon as global radiation goes up in spring, the benefit of the capital stock gets higher. Due to the quadratic investment costs, however, high maintenance investments at once are expensive. Therefore, maintenance investments slowly increase already in winter and reach a peak in spring to have the capital in best condition during summer where global radiation reaches its maximum. This can be seen in Fig. 3b. Further on, renewable energy generation increases in this period and less fossil energy is needed to cover the demand, shown in Fig. 3c. Over summer, investments decline again as the need for maintenance gets lower and they reach their minimum in autumn just before they start to increase again in preparation for the next year. One can see that the optimal investment decision exhibits the same seasonality as the global radiation but it is shifted along the time axis by the expenditure of time for maintenance activities, so that the fluctuation of the capital stock coincides with the one of global radiation.

Such seasonal maintenance planning can be observed in various fields of energy generation. For hydro storage power stations, for example, the seasonality is given by the natural inflows, which usually are higher in early spring due to snow melting and lower over summer during droughts. Also here, maintenance activities are mainly done during the less productive period in summer, in order to keep opportunity costs of a reduced machine availability low. Also thermic power plants usually have their revisions during periods of reduced operating hours due to a lower energy demand (e.g. summer).

Summing up the obtained solutions, we have two periodic solutions being of saddle-type whose areas of attraction probably are separated by an indifference threshold point induced by the unstable focus in between. Indifference threshold points are points in the state space at which the paths leading into different optimal long-run solutions have the same objective value. Therefore, at these points one is indifferent between the two solutions. For more details on indifference threshold points (see Grass et al. 2008; Kiseleva 2011; Kiseleva and Wagener 2010).

### Calculation of the indifference threshold point

**Fig. 4:**
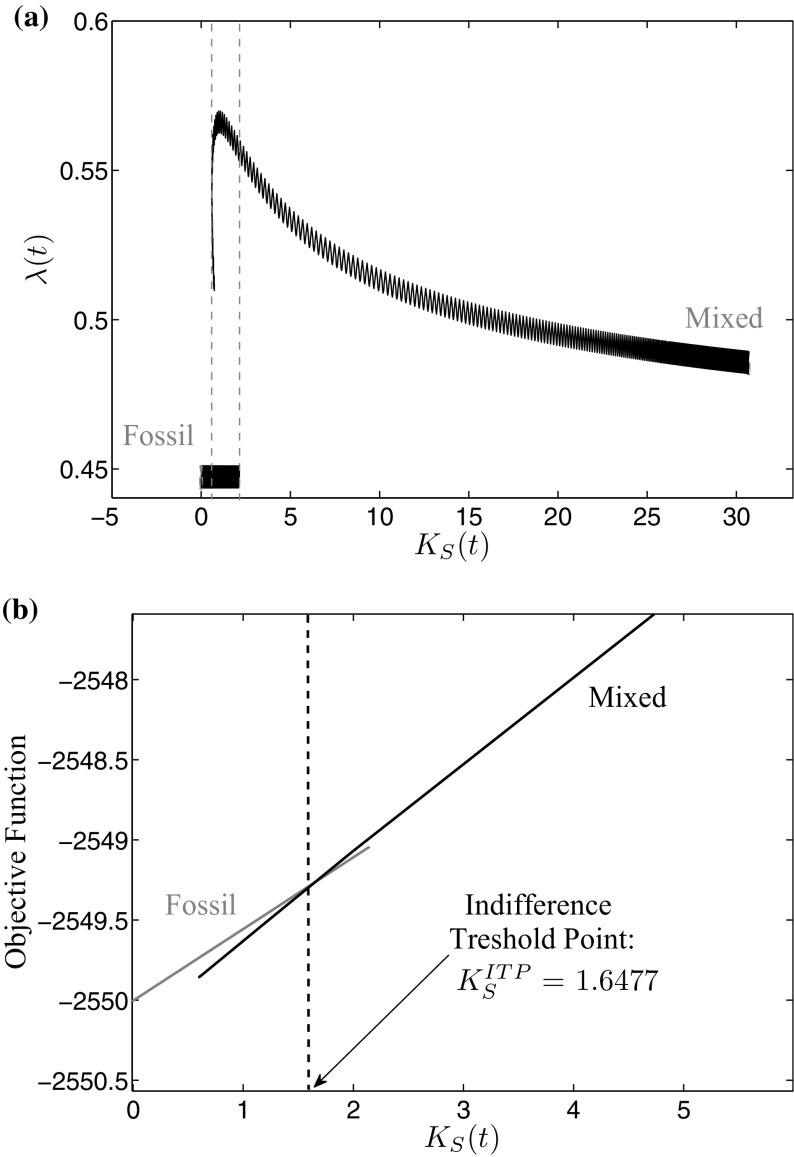
**a** Overlap of trajectories leading into the two periodic solutions and **b** Indifference threshold point: intersection of the objective function values

Whether such an indifference threshold point exists or one of the two periodic solutions being of saddle-type is dominant has, therefore, to be analyzed. To do so, we continue the trajectories of both periodic solutions as far as possible until one of the subsequent cases occurs: (1) the continuation process aborts as the path reaches some feasible boundary, (2) the path is bending back, or (3) the other periodic solution is reached. The results of these continuations can be seen in Fig. 4a. The path starting at the high mixed periodic solution is bending back while the one starting at the fossil periodic solution gets infeasible at some point. To find the indifference threshold point, the objective function values along the two paths are compared to observe whether there exists an intersection. As the analyzed model is non-autonomous, however, the comparison of the objective function values is not time invariant. Therefore, not the objective function values along the last paths of the continuation processes but the last objective values of the paths at each continuation step for the current state value have to be considered. The objective value curves for the two periodic solutions are shown in Fig. 4b. The intersection yields the indifference threshold point, which for the current parameter set lies at $K_{S}^{\mathrm{ITP}} = 1.6477$.

**Fig. 5:**
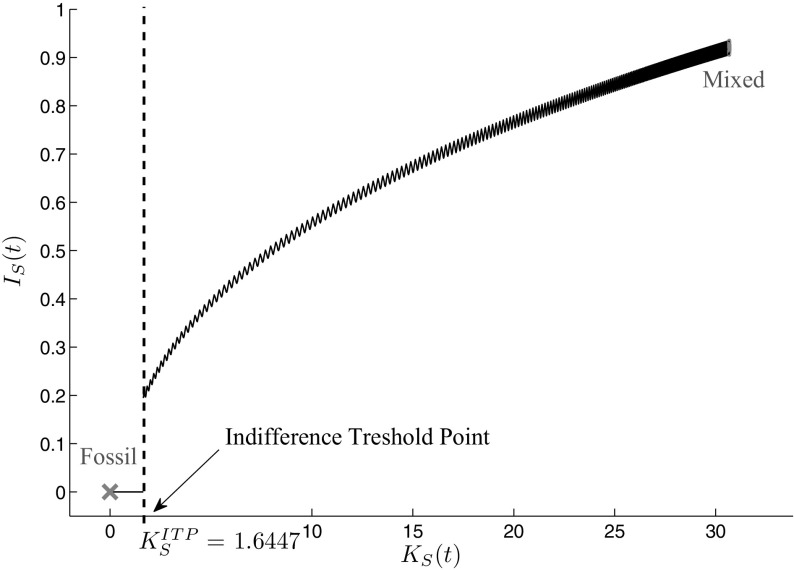
Indifference threshold point and the separated areas of attraction of the two periodic solutions

### Economic interpretation of the indifference threshold point

The occurrence of an indifference threshold point is an important result of this analysis as the optimal long-run periodic solution depends on the initial capital stock with which optimization is started.

Figure 5 shows how the indifference threshold point separates the areas of attraction of the mixed and the fossil periodic solution. If the initial capital stock exactly lies on the indifference threshold point $K_{S}^\mathrm{ITP}$, the paths to both periodic solutions are equally expensive. Therefore, the decision maker is indifferent between increasing investments $I_{S}(t)$ and moving towards the mixed periodic solution with a higher capital stock and a lower fossil energy amount during the summer period on the one hand, and stopping investments and moving towards the fossil periodic solution where all the energy demand is covered with fossil energy on the other hand. If the initial capital stock is higher than the indifference threshold point $K_{S}^{\mathrm{ITP}}$, it is optimal to move up towards the mixed periodic solution, if it is lower, the fossil long-run periodic solution is optimal. The reason for this change lies within the learning-by-doing effect. If the initial capital stock is high enough, the reduction of the investment costs due to the learning-by-doing effect can compensate the cost of additional capital accumulation and, therefore, it is optimal to increase the capital stock, which even enforces this effect but at a decreasing rate. If, however, the initial capital stock is low, the learning-by-doing effect on the investment costs is too weak to compensate the costs for additional capital accumulation. Therefore, it is profitable to reduce investments and, hence, the capital stock, and increase the share of fossil energy used to cover the energy demand until finally, the fossil optimal long-run periodic solution is reached. This initial state-dependent separation of the areas of attraction is also known as history dependence, as the optimal long-run periodic solution is determined by the accumulation effort for renewable energy capital in the past.

This result points out the difficulty of introducing a new energy technology into the market. While conventional energy types already are competitive and have low prices due to the high experience accumulated over years, the investment costs for new technologies are very high. As no experience exists at the beginning, these high investment cost would have to be paid over some period of time during which the new technology definitely is not profitable, until finally at least some reduction due to accumulated experience is archived which would be the very first step on the long way towards the break even point. This aspect underlines the importance of subsidies and other kind of financial support that is necessary during the starting-up period to help new technologies over this barrier. Such temporary incentives, also known as directed technical change, can be fundamental for the inclusion of clean technologies. See for this Acemoglu et al. (2012), where it is shown that for an economy consisting of a clean and a dirty technology being sufficiently substitutable, environmental regulation is essential to avoid an environmental disaster. As soon as the clean technology is adequately advanced, however, no further regulation is needed anymore as profit-maximizing production will automatically shift to the clean technology. As in contrast to this, our model approach does not consider such regulations, it, therefore, would never be optimal to start with the renewable energy technology from the very beginning. If no experience exists to reduce the initially high investment costs, fossil energy always is less cost intensive and, as no further restrictions are included like CO$_{2}$ performance standards for example, no switch to a cleaner energy technology would happen. Only, if there is already a sufficiently high level of experience when optimization is started, further investments are profitable.

### Breakeven analysis

As accumulated experience improves the technical processes and hence reduces the necessary financial effort, the technology gets more profitable. However, it can take a long time until full competitiveness with the conventional technology is achieved, which happens at the so-called break-even point.

**Fig. 6:**
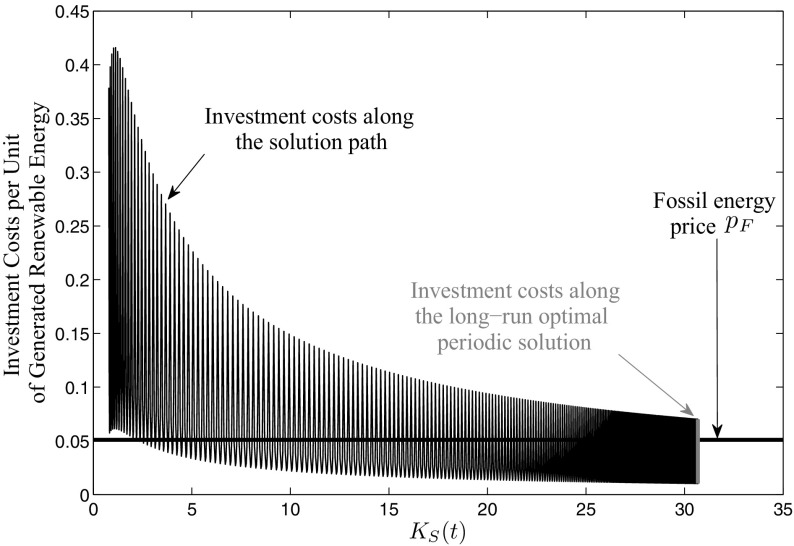
Investment costs per unit of generated renewable energy along the path leading into the mixed optimal long-run periodic solution

To analyze the extend of the learning-by-doing effect on the investment costs in our model, we compare the costs of renewable energy generation with the fossil energy price ${{ p}_{ F}}$ along the path leading into the optimal long-run periodic solution. The investment costs per unit of generated renewable energy at this time is given by the term

25 $$\begin{aligned} \frac{\left( b I_{S}^{*}(t)+c I_{S}^{*}(t)^{2}\right) (K_{S}^{*}(t)+\epsilon )^{-\alpha }}{(\nu \sin ^{2}(t\pi )+\tau \big ) K_{S}^{*}(t) \eta }, \end{aligned}$$

where $K_{S}^{*}(t)$ and $I_{S}^{*}(t)$ are the state and the control along the path leading into the optimal long-run periodic solution. The results can be seen in Fig. 6. As the generation of renewable energy fluctuates in time with the available global radiation and occurs in the denominator of Eq. (25), the investment costs also vary over the period. However, a clear decreasing tendency can be observed as soon as capital is accumulated. The black horizontal line in Fig. 6 shows the fossil energy price ${{ p}_{ F}}$. At the beginning of the path, the investment costs are very high, especially in winter they are almost the eightfold of the fossil energy price ${{ p}_{ F}}$. The reasons for this are the initially high investment costs of the renewable energy technology together with the low initial capital stock and, hence, the low amount of generated renewable energy. In summer, however, one can see that the investment costs are lower because global radiation is high and, therefore, more renewable energy is generated. Very early along the path even the fossil energy price level is reached during summer. As the path proceeds, investments accumulate new capital and, therefore, the learning-by-doing effect as well as the generated renewable energy increases. This leads to declining price levels, both in winter and summer, and also the margin between these two decreases until finally the optimal long-run periodic solution is reached. Here, the price level in summer is already far below the fossil energy price level while in winter it is still above it. However, over the year the benefit of the portfolio mixture is high enough to let the combination of fossil and renewable energy be optimal.

## Sensitivity analysis

The analysis of the previous section has shown that the learning-by-doing effect can imply history dependence of the optimal long-run periodic solution. The driving force for this dependence is given by the cost-effectiveness of renewable energy generation with respect to conventional energy technologies. However, there are several factors beyond historical capital accumulation activities that influence this cost-effectiveness. First of all, of course, the fossil energy price ${{ p}_{ F}}$ plays a major role, reflecting the economic performance of the fossil technology. Further on, it is essential how strong the cost decreasing influence of the learning-by-doing effect is on the investment costs of renewable energy. Besides that, also the performance of the renewable energy generation is important, which is determined for example by site-specific factors as for example the supply of global radiation. To analyze how the obtained results of the previous section change when these factors vary, we conduct in this section a sensitivity analysis with respect to the fossil energy price ${{ p}_{ F}}$, the learning coefficient $\alpha$, and different sets of the parameters $\tau$ and $\nu$ that determine the site specific global radiation intensity.

**Fig. 7:**
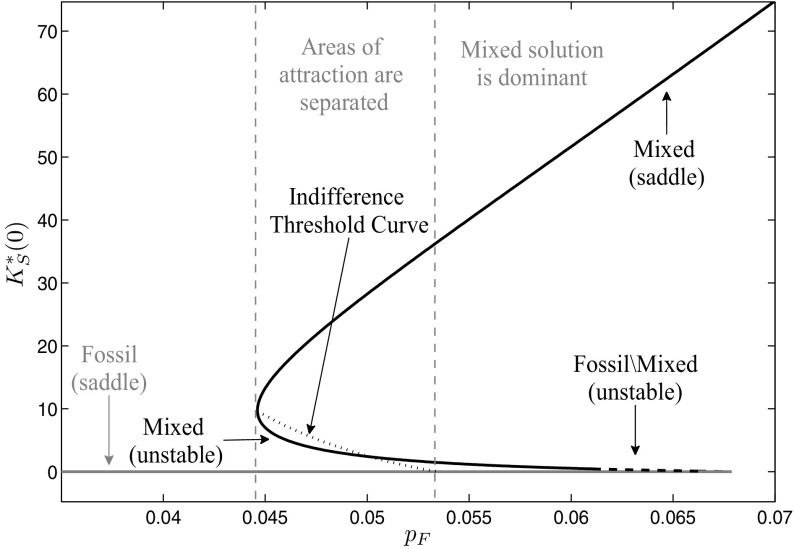
Bifurcation diagram with respect to a fossil energy price ${{ p}_{ F}}\le 0.07$

### Fossil energy price ${{ p}_{ F}}$

**Fig. 8:**
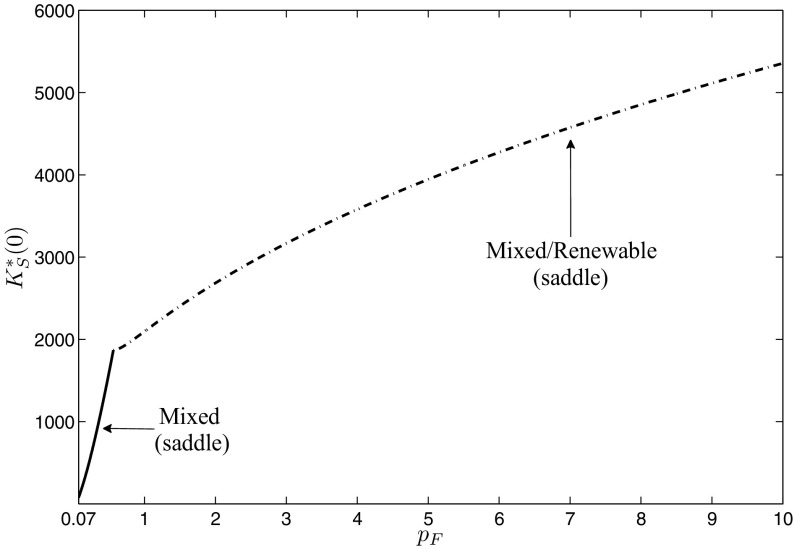
Bifurcation diagram with respect to a fossil energy price ${{ p}_{ F}}\ge 0.07$

**Fig. 9:**
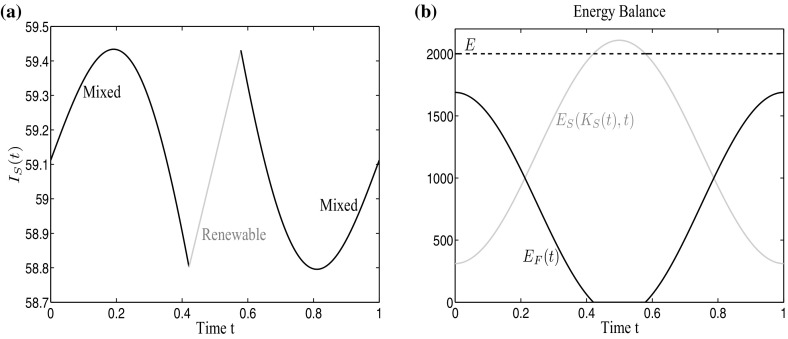
Mixed-arc solution with two mixed arcs and one renewable arc in between: **a** Investment $I_{S}(t)$ and **b** energy balance

In the first step, we focus on the influence of the fossil energy price on the optimal portfolio composition. We use numerical continuation with respect to the fossil energy price ${{ p}_{ F}}$ to investigate how the results change when fossil energy gets more expensive. Note that we always consider in the following the bifurcation of the canonical system, not of the optimal system. Therefore, also the changes in the unstable as well as the dominated long-run periodic solutions are shown. The results can be seen in Fig. 7, where the starting points $K^{*}_{S}(0)$ of the periodic solutions are plotted as gray line for the fossil solution and as black line for the mixed solutions. If the fossil energy price is very low, there exists only the fossil periodic solution because the opportunity costs of investments into renewable energy capital are so high that they are not profitable and, hence, no investments at all are done and the whole energy demand is covered only with fossil energy. Starting at a fossil energy price of ${{ p}_{ F}}=0.0446$, there exists also the two mixed periodic solutions, where the lower one is unstable and the upper one is of saddle-type. The areas of attraction of the fossil and the upper mixed periodic solutions are separated by indifference threshold points summarized in the indifference threshold curve plotted as black dotted line. At the beginning, it lies above the unstable mixed long-run solution. As fossil energy in this area still is comparatively cheap, the historical renewable energy capital accumulation efforts have to be very high to make further investments in renewable energy capital profitable. If the fossil energy price further increases, the indifference threshold curve declines as renewable energy capital investments are profitable already at a lower historical capital accumulation effort. At ${{ p}_{ F}}=0.0501$, the indifference threshold curve intersects with the mixed unstable long-run periodic solution. From then on, the areas of attraction are separated below this periodic solution. At ${{ p}_{ F}}=0.0535$ it ends at the fossil periodic solution. For a fossil energy price $0.0535\le {{ p}_{ F}}\le 0.0679$, still all three long-run periodic solutions exist, but the high mixed one is dominant as here fossil energy alone would be too expensive to cover the demand. The unstable mixed solution turns into a multi-arc solution with two mixed arcs and one fossil arc in between at ${{ p}_{ F}}=0.0612$, as investments decline with the fossil energy price until they finally get zero. This first happens during the winter period while during the summer period investments are still positive. As one can see in the figure, the fossil solution only exists to some specific fossil energy price. The reason for this is that the Lagrange multiplier $\mu _{3}(t)$ becomes negative. The price at which this happens is a function of state $K_{S}(t)$ and time *t* and for the current parameter set and the fossil solution is given as ${{ p}_{ F}}=0.0678$. For higher values of ${{ p}_{ F}}$, however, a fossil-mixed solution still can be feasible if the part along which the Lagrange multiplier would be negative is replaced by a mixed arc. As soon as the Lagrange multiplier is negative already at the point of time where $\lambda (t)$ reaches its minimum, however, also no feasible fossil-mixed solution exists, which is for the current parameter set at ${{ p}_{ F}}=0.069$. For fossil energy prices ${{ p}_{ F}}{>}0.069$, the optimal long-run periodic solution is given by the high mixed periodic solution. Figure 8 shows what happens if the fossil energy price ${{ p}_{ F}}$ increases even beyond 0.07. As renewable energy generation progressively gets profitable due to the reduced investment costs by the accumulated experience as well as the comparatively more expensive fossil energy, a strong increase in renewable energy generation capital can be observed. However, still both energy types are needed over the whole period to cover the given energy demand. At ${{ p}_{ F}}=0.5613$, renewable energy generation capital is so high that during summer, when global radiation reaches its maximum, the demand even can be covered without fossil energy. At this point, the feasible boundary of the mixed case is reached and, from this fossil energy price on, a periodic solution exists that consists of two mixed arcs and a renewable arc in between. Figure 9 shows such a mixed/renewable solution in more detail for a fossil energy price of ${{ p}_{ F}}=0.8$. Along these mixed/renewable solutions, the demand over some time interval in summer is covered only by renewable energy, while in winter fossil energy still is needed in addition. If the fossil energy price increases even more, there is still an increase in the stock of renewable energy capital, however, obviously at a decreasing rate. The reason for this is that the marginal benefit of an additional unit of renewable energy capital declines. Remember that generated surpluses beyond the energy demand cannot be used because storage is not included in the model. Therefore, a further increase of the capital stock only is profitable along the mixed arcs, where the necessary amount of fossil energy can be reduced by slightly increasing renewable energy generation. But as the global radiation at the switching times between the arcs gets lower, the closer they are to 0 and 1, more and more renewable energy capital is necessary to compensate. Although the investment costs of renewable energy capital decline with the increasing capital stock and reduce at least the financial effort for this compensation, this saturation effect occurs.

**Fig. 10:**
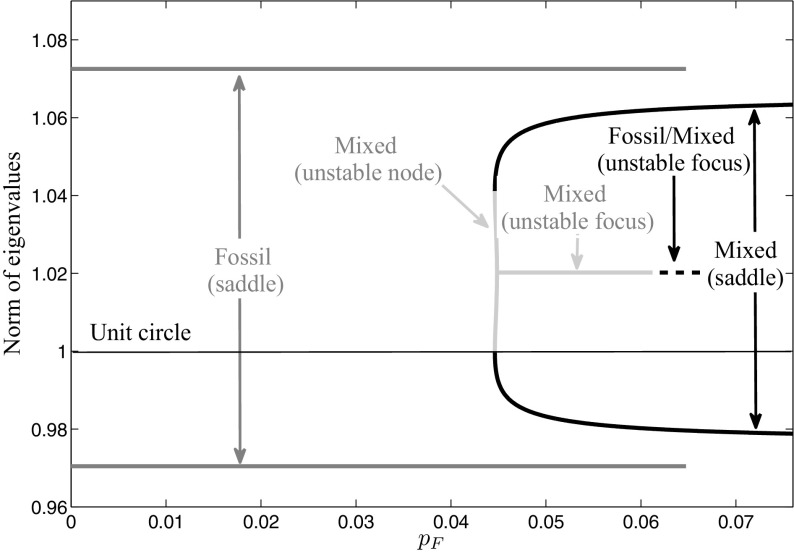
Norm of eigenvalues of long-run periodic solutions for a fossil energy price ${{ p}_{ F}}\le 0.07$

Figure 7 further on shows that a turning point occurs at ${{ p}_{ F}}=0.044$ in the mixed solution. To investigate how the optimal vector field changes here, we consider the local behavior of the monodromy matrix. Figure 10 shows the norm of the eigenvalues of each long-run periodic solution along the ${{ p}_{ F}}$-axis. The eigenvalues belonging to the fossil long-run periodic solution are shown in dark gray. As we already have shown in Sect. 4.3, the monodromy matrix and hence the eigenvalues of any fossil solution are independent on the periodic solution itself as no state nor co-state occurs in the Jacobian for this case. Hence, the eigenvalues of the fossil long-run periodic solution in Fig. 10 are constant over the fossil energy price ${{ p}_{ F}}$ and are given as $e_{1}=e^{-\delta _{S}},~e_{2}=e^{r+\delta _{S}}$. As one eigenvalue lies within and the other one outside the unit circle, which in the figure is plotted as black horizontal line, the fossil solution is of saddle-type over its whole interval of existence. The probably most interesting result can be observed for the mixed solutions. The eigenvalues corresponding to the upper mixed long-run periodic solution are shown in Fig. 10 as black line, where again one is lying within and the other one outside the unit circle which specifies the solutions to be of saddle-type. The lower the fossil energy price ${{ p}_{ F}}$, the higher gets the stable eigenvalue until finally, at ${{ p}_{ F}}=0.044$, it crosses the unit circle. Hence, a fold-bifurcation occurs (see for more details Reithmeier 1991) and the stability of the mixed long-run periodic solution changes from saddle-point stability to unstable. The two eigenvalues outside the unit circle are plotted as light gray lines in Fig. 10. At the beginning, they are still real and, hence, the lower mixed periodic solution is an unstable node, but very soon they get complex and the mixed periodic solution turns into an unstable focus. At ${{ p}_{ F}}=0.0612$, the lower mixed periodic solution merges into the fossil-mixed solution whose eigenvalues are shown as light gray dotted line. Also here, the eigenvalues are complex and their real parts are outside of the unit circle, which specifies this solutions as unstable focus as well.

### Learning coefficient $\alpha$

As already mentioned, not only the fossil energy price plays an important role how the optimal portfolio composition looks like, but also the reducing impact of the learning-by-doing effect on the investment costs of renewable energy, which is determined by the learning coefficient $\alpha$. In literature, many research papers can be found that investigate the correct height of learning coefficients for different types of technologies. However, opinions strongly differ. To analyze how sensitive the optimal portfolio composition is to different assumptions on the learning coefficient, we conduct in this section the same analysis as in the previous one, but this time with respect to the learning coefficient $\alpha$.

**Fig. 11:**
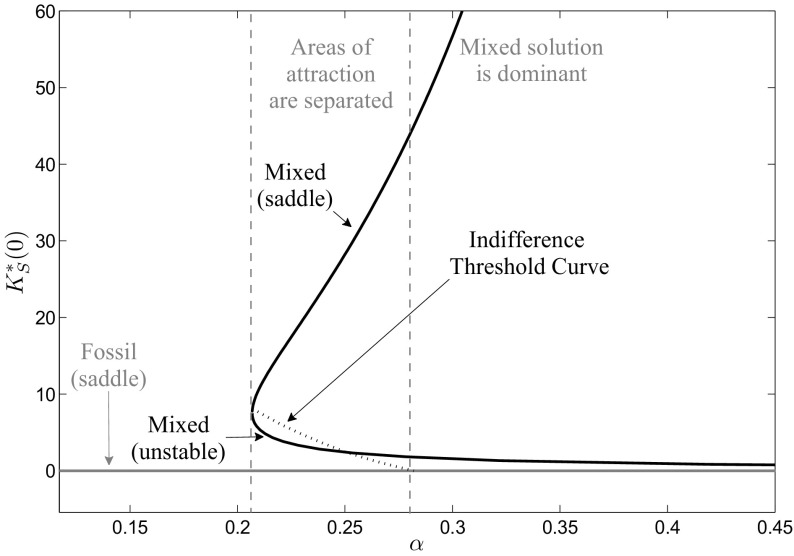
Bifurcation diagram with respect to the learning coefficient $\alpha$

We fix the fossil energy price at ${{ p}_{ F}}=0.05$ and again use numerical continuation to calculate the optimal long-run periodic solutions as well as the indifference threshold points, if existent, for a varying $\alpha$. The results can be seen in Fig. 11. For a learning coefficient of $\alpha <0.2068$, which corresponds to a learning-by-doing rate of $\mathrm{LDR}<13.35\,\%$, the optimal long-run periodic solution is given by the fossil periodic solution. The reason for this is the aspect that the learning-by-doing effect is too weak to compensate the initially high investment costs to make it profitable to invest in renewable energy generation capital and, hence, the whole demand is covered with fossil energy. For learning coefficients close to $\alpha >0.2068$, three long-run periodic solutions exist of which one is the fossil solution and the other two are the two mixed solutions where the higher one is of saddle-type and the lower one is unstable. Indifference threshold points separate again the areas of attraction. The economic interpretation of this result is that the historical renewable energy capital efforts that are necessary to make renewable energy investments profitable decline with the intensity of the learning-by-doing effect, as a lower initial renewable energy capital stock then already is sufficient. Until $\alpha =0.2505$, which corresponds to a learning-by-doing rate of $\mathrm{LDR}=15.94\,\%$, the indifference threshold curve lies beyond the unstable mixed solution. Also here, the path leading into the periodic solution has to be continued to a mixed arc path to get the indifference threshold point. For $\alpha >0.2505$, the indifference threshold curve lies below the unstable mixed solution and further declines in $\alpha$ until finally, at $\alpha =0.282$ and, hence, at a learning-by-doing rate $\mathrm{LDR}=17.75\,\%$, it coincides with the unstable mixed solution. For higher learning coefficients, the mixed periodic solution dominates the fossil one as fossil energy is too expensive to be exclusively used to cover the demand.

### Global radiation intensity

So far we have investigated the impact of price and learning-by-doing effects on the optimal portfolio composition. However, we completely have fixed site-specific aspects concerning the supply of global radiation for the previous analysis. Therefore, an interesting aspect on which we focus on in the following is as to how the solutions change when geographical conditions vary.

**Fig. 12:**
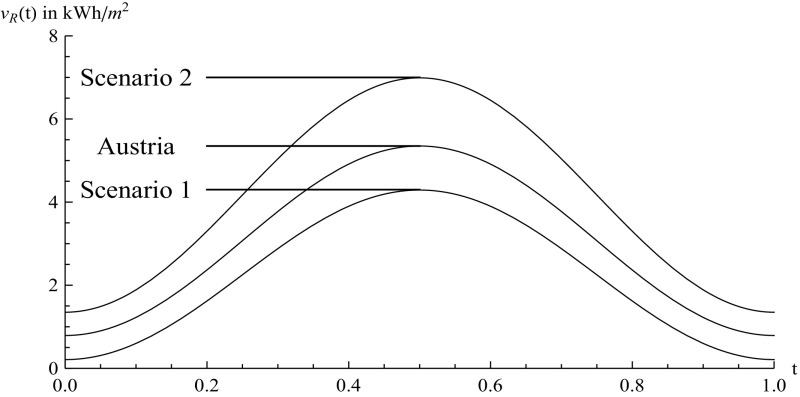
Deterministic functions for global radiation

For the estimation of the parameter values $\tau$ and $\nu$ for the analysis so far, we have used Austrian data. However, as the global radiation intensity strongly varies with the geographical position, the question rises how the results would change if data for a geographical site higher in the north or lower in the south are used instead? To do so, we use global radiation data for Hamburg (Scenario 1) and from Athens (Scenario 2) as examples for a more northern and a more southern site, respectively (source of data see SODA 2014). The results of the parameter estimations are summarized in Table 3 and the deterministic functions for Scenarios 1 and 2 and also the original estimates for Austria, which we already have used for the previous analysis, are shown in Fig. 12.

**Table 3:** Estimates for $\tau$ and $\nu$

	$\tau$	$\nu$
Austria	0.79	4.56
Scenario 1	0.21	4.08
Scenario 2	1.35	5.64

In order to investigate the changes in the optimal portfolio composition when site-specific parameters change, we conduct the same sensitivity analysis with respect to the fossil energy price ${{ p}_{ F}}$, as done in Sect. 6.1, and compare the different outcomes.

### Sensitivity analysis for Scenarios 1 and 2

**Fig. 13:**
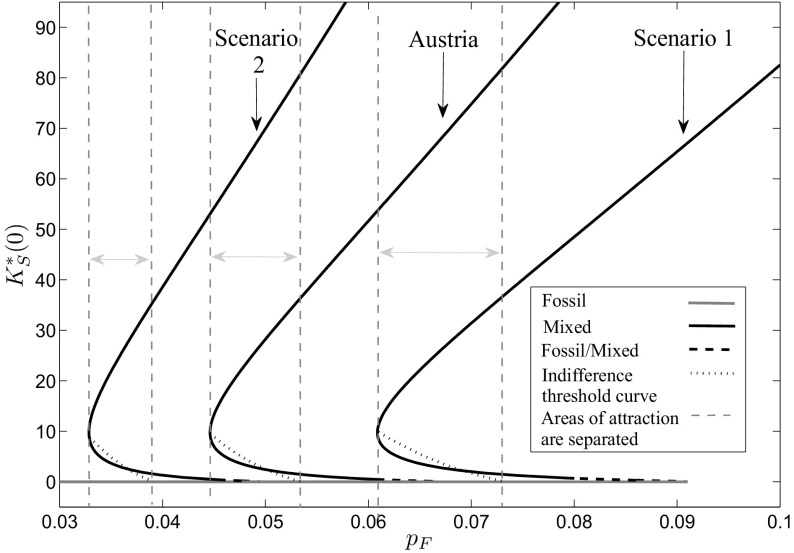
Bifurcation diagram with respect to the fossil energy price ${{ p}_{ F}}$ for the Scenarios 1 and 2 in comparison with the results for Austria

Figure 13 shows the results of the sensitivity analysis for Scenarios 1 and 2, respectively, compared to the results we have obtained for the parameters estimated for Austria.

First, we focus on Scenario 1 with a less intensive supply of global radiation. It shows that the qualitative behavior is the same. For a low fossil energy price only the fossil solution exists, while at a specific point the two mixed solutions, with one being unstable and the other one being of saddle-type, occur and the areas of attraction are separated by an indifference threshold curve. However, a look on the price axis makes clear that remarkable changes concerning the position have happened. While the first bifurcation point at which these two additional mixed periodic solutions exists has been at ${{ p}_{ F}}=0.0446$ for the original set, this happens here at ${{ p}_{ F}}=0.0609$. Although the intensity of the learning-by-doing effect is the same and, therefore, the investment costs per unit capital would decline at the same speed, the lower global radiation supply leads to a lower renewable energy generation and, hence, to higher investment costs per unit of power. This aspect shifts the interval in which the mixed periodic solutions as well as the indifference threshold curve exist to the right as the fossil energy price has to be much higher to make further investments profitable. Consequently, also the price level at which the high mixed solution gets dominant and, hence, the pure fossil solution is not further optimal, has shifted to the right. For the original parameter set this happens at ${{ p}_{ F}}=0.0535$, while here the price level for this is much higher at ${{ p}_{ F}}=0.0739$. Finally, at ${{ p}_{ F}}=0.091$ the optimal long-run periodic solution is given by the high mixed periodic solution. Furthermore, the slope, with which the high mixed periodic solution increases with the fossil energy price, is lower compared to the basic scenario for Austria. The reason for this is given by the fact that, due to the lower global radiation, less renewable energy can be generated and, hence, the optimal renewable energy capital stock is lower at the same fossil energy price. Additionally, one can see that also the interval gets larger in which the indifference threshold curve separates the areas of attraction of the two periodic solutions being of saddle-type. This is because also the capital stock, at which the mixed periodic solution starts to dominate the fossil one, is reached at a higher fossil energy price.

Second, we investigate Scenario 2 with a higher intensity of global radiation. Also for this case, the qualitative outcome does not change, but again the price boundaries are of special interest. While the interval, in which all three long-run periodic solutions exist and the area of attraction is separated by an indifference threshold point, started at ${{ p}_{ F}}=0.0446$ in the original set and at ${{ p}_{ F}}=0.0609$ in Scenario 1, one can observe from Fig. 13 that this here happens already at ${{ p}_{ F}}=0.0328$. As the supply of global radiation is higher, the investment costs per unit of power for an equal capital stock here are even lower than for the other two cases. Hence, investments into renewable energy get profitable already at a lower fossil energy price. For this reason, also the indifference threshold curve has shifted to the left. The high mixed solution in Scenario 2 gets dominant at ${{ p}_{ F}}=0.0449$, a price at which in the original set a mixed portfolio just starts to be an alternative to the pure fossil one, not to mention Scenario 1 where this possibility does not exist at all at this price level. Starting at ${{ p}_{ F}}=0.0495$, the high mixed solution is the optimal long-run periodic solution. Here, the slope, with which the high mixed periodic solution increases with the fossil energy price, is higher compared to the basic scenario for Austria. Due to the higher global radiation, more renewable energy can be generated and, hence, a higher renewable energy capital stock is profitable already at a lower fossil energy price. Consequently, the interval, in which the indifference threshold curve separates the areas of attraction of the two periodic solutions being of saddle-type, gets smaller as the capital stock, at which the mixed periodic solution with research starts to dominate the fossil one, is reached at a lower fossil energy price.

Varying the intensity of the site-specific global radiation has shown some interesting aspects. While in all three cases, the original parameter set as well as the two scenarios, the intensity of the learning-by-doing effect is exactly the same, the outcomes and their possible consequences for political decisions are completely different. In reality, of course, the price boundaries between which the indifference threshold curve separates the areas of attraction are hard to observe and the only indicator for subsidy decisions might be given by the current fossil energy price. Assume, that in all three cases a price level of ${{ p}_{ F}}=0.05$ is given and subsidies are set to foster renewable energy generation. While for the original parameter set at this price level indeed the indifference threshold curve occurs and the subsidies could help to achieve a switch to renewable energy generation, for the southern country in Scenario 2 the mixed portfolio is already the only optimal solution at this price. Consequently, renewable energy generation here would be over subsidized. Otherwise, for the northern country in Scenario 1, renewable energy generation is not at all an option at this price level as the fossil solution is the only optimal solution and the subsidies here would be completely ineffective. This shows how sensitive the effectiveness of subsidies is to country-specific conditions. Same would also apply for taxes on fossil energy as the shift in the fossil energy price, that is necessary to enable a switch to renewable energy generation, depends on the country-specific situation as well.

## Conclusions

We have investigated in this paper how a small country’s optimal composition of a portfolio consisting of fossil and renewable (solar) energy looks like, when the effect of learning by doing reduces the investment costs due to accumulated experience. Modeling the problem as a non-autonomous optimal control model, we have included a one-factor log-linear learning curve into the objective function so that the accumulated renewable energy capital, which is supposed to reflect the collected experience, has a diminishing impact on the investment costs. Further on, we postulated seasonally varying renewable energy supply and a well-known energy demand that has to be covered.

The obtained results have shown that the fluctuating supply of the renewable resource is one of the major challenges for renewable energy generation. Even if the break even point for the renewable energy technology could be reached where it gets competitive, the shortfalls in winter still have to be covered with fossil energy. This, however, implies another problem which can be seen at the current market situation. As no resource costs occur for renewable energy generation, the variable costs are almost zero, well in contrast to fossil energy. Therefore, a too high renewable energy generation could jeopardize the competitiveness of fossil energy and, hence, its capability to cover these shortfalls. However, as long as the renewable energy generation is not autarkic, for example due to the help of supportive storage systems, fossil energy is urgently needed as backup.

Sensitivity analysis with respect to the fossil energy price ${{ p}_{ F}}$ has shown that there exist price intervals in which multiple periodic solutions occur, and whose areas of attraction are separated by an indifference threshold point. Further on, it turns out that these results are not only sensitive to the fossil energy price but also to the intensity of the learning-by-doing effect as well as on geographical conditions concerning the global radiation.

The occurrence of an indifference threshold point yields important aspects for the economic interpretation of the obtained results. We have seen that whether investments into renewable energy generation capital are worthwhile or not depends on the initial capital stock. Due to this history dependence, investments into renewable energy generation from the very beginning never would be optimal in our approach as the initial investment costs are too high. The level of the capital stock, at which such investments get profitable, shifts even further up if global radiation is lower, as for the northern countries, or if the learning-by-doing effect is weaker, meaning that the learning coefficient is assumed to be lower. One important conclusion of these results is that financial support in form of subsidies during the starting up period of a new technology could play a major role for the successful introduction of this technology into the market. The profitability, however, strongly depends on the site-specific conditions.

The most important aspect for an increasing renewable energy generation in our model has been given by accumulated experience. This learning-by-doing effect, however, has been restricted to the considered country itself. In reality, of course, learning by doing is not only a national but also an international issue. Knowledge spillovers between different countries would even enforce the learning effect and, hence, the adaption of renewable energy technology would happen much faster. Synergies between different countries, therefore, could shift the indifference threshold point to the left and, consequently, a lower subsidy effort would be necessary to support the inclusion of renewable energy generation into the portfolio.

While international cooperation can be supportive for accumulating experience with renewable energy generation, the results from the sensitivity analysis with respect to the global radiation intensity have shown that the effectiveness of a subsidy system strongly depends on national conditions. This means that a cross-border subsidies policy might overlook such tiny but important differences between countries, which could make the subsidies ineffective, as we have seen in Sect. 6.3, and, therefore, could be counterproductive. Consequently, this implies that subsidy policies should remain a national issue while international cooperation and knowledge exchange on renewable energy technologies should be fostered.

Experience in this approach has been the driving force for the reduction of investment costs. But this is not the only source for technological learning. Of course also research and development efforts could foster the competitiveness of a new technology, which implies accumulation of knowledge and, hence, an additional reduction in investment costs. The extension of the model with this aspect will be of special interest in one of our future works.

## Footnotes

1 Remember that, for simplicity, the energy demand is assumed to be constant and stationary. Therefore, here no time argument appears. We also have analyzed similar approaches with a seasonally fluctuating energy demand (peaks in winter and/or summer due to heating and air conditioning), but comparing the results has shown that the observed effects remain the same, only the quantitative portfolio composition might change a little bit. For more details about the different model approaches that we have considered see also Moser (2014).

2 In practice, of course, small surpluses generally would be traded on the market. However, in times of great surpluses as it sometimes occurs around Christmas, prices often turn negative which also comes along with great losses. Therefore, we do not further include the trading aspect in our model but consider such losses in form of sunk investment costs.

3 Note that from here on we often omit the time argument in the function arguments for the ease of exposition.

4 Note that for the fossil case the generated renewable energy $E_{S}(t,K_{S}(t))$ still is included in the energy balance equation. This is because renewable energy at the beginning of the path could still contribute to the portfolio if there is an initially positive capital stock. As no further investments are done, however, the capital stock will decline over time and the contribution of renewable energy gets negligibly small in the long-term. If, in contrast, the initial capital stock is zero, the contribution is zero along the whole path.

5 For discounted optimal control problems ($r>0$), unstable solutions are the only possible limit sets of the canonical system. Consequently, solutions of saddle-type and their stable paths are considered to be the most important candidates for optimal solutions of such problems, in case it is assumed that an optimal solution converges to its limit set. For more details on this see Feichtinger and Hartl (1986) and Grass et al. (2008).
